# Recruitment of Participants for a 3D Virtual Supermarket: Cross-sectional Observational Study

**DOI:** 10.2196/19234

**Published:** 2021-02-09

**Authors:** Jody C Hoenink, Joreintje D Mackenbach, Laura Nynke van der Laan, Jeroen Lakerveld, Wilma Waterlander, Joline W J Beulens

**Affiliations:** 1 Department of Epidemiology and Data Science Amsterdam UMC, Vrije Universiteit Amsterdam Amsterdam Public Health Research Institute Amsterdam Netherlands; 2 Department of Communication and Cognition Tilburg School of Humanities and Digital Sciences Tilburg University Tilburg Netherlands; 3 Department of Public Health Amsterdam UMC, University of Amsterdam Amsterdam Public Health Research Institute Amsterdam Netherlands; 4 Julius Center for Health Sciences and Primary Care University Medical Center Utrecht Utrecht University Utrecht Netherlands

**Keywords:** online study, nudges, pricing, recruitment strategies

## Abstract

**Background:**

Virtual supermarkets offer a practical and affordable setting to test the efficacy of different pricing and nudging strategies before they are implemented in the real world. Despite the advantages of using virtual supermarkets for this purpose, conducting studies in online settings is challenging with regard to recruitment and retention of sufficient and suitable participants.

**Objective:**

To describe cost, time, and retention with regard to participants recruited using various strategies and potential sociodemographic differences between participants recruited via different strategies.

**Methods:**

This cross-sectional study used data from a randomized controlled trial in which 455 Dutch adults with low and high educational levels were invited to shop 5 times in a 3D virtual supermarket. Participants were recruited via social media and flyers. A log that tracked the costs of and time spent on the different recruitment strategies was kept by the study team. Outcome measures included the cost of recruitment strategies, the time investment by researchers, and recruitment and attrition rates of participants in the study.

**Results:**

The median age of study completers was 31.0 (IQR 25.0) and 157 out of 346 study completers (45.4%) were highly educated. Out of the 455 included participants, 235 (51.6%) were recruited via social media campaigns, 131 (28.8%) via home-delivered flyers, 38 (8.4%) via flyers directly distributed by the study team, and 46 (10.1%) via word-of-mouth. Of all paid recruitment strategies, social media campaigns were the cheapest and least time-consuming, whereas the distribution of flyers by the study team was the most expensive and time-consuming recruitment strategy. Age, sex, overweight status, employment situation, and number of adults within the household varied by recruitment strategy.

**Conclusions:**

Using different recruitment strategies resulted in the efficient recruitment of a representative study sample and retention of participants was relatively high. While “word-of-mouth” was the most cost- and time-effective recruitment strategy, using only one type of recruitment strategy could result in a demographically skewed study population.

## Introduction

Supermarkets are an important point-of-purchase setting [1] particularly applicable in studies targeting diet as a risk factor for noncommunicable diseases. Examples of promising strategies to improve population diets in supermarket settings include pricing and nudging strategies. Studies have shown that pricing (eg, price promotions on healthier products) and nudging (eg, prominent placement of healthier products) strategies can be effective in increasing purchases of healthy foods [2-5]. While pricing strategies can be seen as “harder” approaches, nudges can be seen as “softer,” as nudges are less intrusive and simply alter the choice environment to make the healthy choice the easier choice, without removing the unhealthy choice [3]. Despite their promise, investigating the effectiveness of pricing (especially increasing the price of unhealthy foods) and nudging strategies in real supermarkets is costly (eg, purchases of materials or compensation of the supermarket for loss of revenue) and time consuming for researchers (eg, recruitment of participants, collecting receipts, imputing purchasing data).

Virtual supermarket environments may offer a practical and affordable means of testing the efficacy of different pricing and nudging strategies *before* they are implemented in real-world settings. Virtual supermarket environments include online web shops for grocery shopping and 3D virtual supermarkets. The 3D virtual supermarket imitates a real-life supermarket by duplicating the layout and using 3D products. Once a virtual supermarket is constructed, researchers can easily manipulate the supermarket environment by adjusting prices, and adding nudges such as posters or frames around products. A number of 3D virtual supermarkets have been developed to date [6-9]. Overall, previous studies of 3D virtual supermarkets have indicated that they are a valid tool for investigating the effect of pricing strategies on food purchases [6], also when compared to real-life purchases [8]. As such, 3D virtual supermarkets appear to be a valid alternative to real-world supermarkets as an environment in which the efficacy of nudging and pricing strategies can be studied.

Recruitment of a sufficiently large sample that adequately represents the target population can be difficult [10]. Reporting on the effectiveness of recruitment strategies facilitates improvements in the design and methods of future studies. The effectiveness of recruitment strategies depends on several factors including the study design, setting, study population, and the use of incentives [11]. Despite the advantages of 3D virtual supermarkets, it may be more difficult to recruit participants for online studies compared to interventions in real-world settings, as participants need to be adept at using technology and need to have access to a smartphone or computer with internet access [12,13]. Difficulty with recruitment can lead to longer recruitment times, increased use of resources, and reduced sample size and power. Additionally, it may be more difficult to retain participants as compared to studies in real-world settings due to the lack of personal contact [11,13] for example, which may lead to selection bias and loss of statistical power [12]. Furthermore, particularly when using a within-subject study design where participants are asked to conduct multiple rounds of shopping over a specified period, long waits between these shopping trips might lead to diminished interest, as the novelty of the online intervention decreases, and increased frustration, resulting in additional attrition. While some degree of attrition is largely inevitable, excessive attrition reduces statistical power, increases bias, and leads to lower generalizability of results [13].

Evidence suggests that most intervention studies, that is, experimental studies online or in the real world in which investigators assign the exposure(s) to participants, use print advertising such as flyers, posters, and newspaper advertisements to recruit potential participants [14,15]. Challenges related to the recruitment and retention of participants in online studies have led to the use of alternative recruitment strategies that rely on internet advertising and social media [13]. These innovative recruitment strategies are attractive due to their potential to reach a larger number of people, apparent cost-effectiveness, and ability to reach populations that are considered hard-to-reach (eg, young adults and adults with a lower educational level) [13,15]. Despite the growing popularity of recruitment via social media, data on the effectiveness of this strategy in the context of online studies are limited [15].

Online studies have reported on the use of several recruitment strategies [6,11]. However, as far as we are aware, no studies investigating the effectiveness of social media as a recruitment strategy have been conducted in the Netherlands to date [13]. The aim of this study was to describe cost, time, and retention rates with regard to different recruitment strategies (including innovative and traditional recruitment strategies), and the sociodemographic characteristics of participants recruited via these different strategies.

## Methods

### Study Overview

This study is part of the “Sustainable Prevention of Cardiometabolic Risk through Nudging Health Behaviors” (Supreme Nudge) project [16]. Data presented in this paper describe the cost, time, and retention rates associated with different recruitment strategies from a larger study investigating the efficacy of nudging and pricing strategies on food purchasing behavior in a virtual supermarket and effect modification by socioeconomic position (SEP). Results of this trial are reported elsewhere [17] and additional details about the study aims and design are presented in Multimedia Appendix 1. The study design and procedures for this virtual supermarket study were approved by the Medical Ethics Review Committee of VU University Medical Centre (OHRP: IRB00002911), and all participants provided informed consent.

### Inclusion Criteria

Inclusion criteria were that participants had to be 18 years or older, were able to communicate in Dutch, had access to a computer with internet, had a valid email address, and regularly did the grocery shopping for their household. This study aimed to include an approximately equal number of lower and higher SEP adults determined using the proxy educational level. Given the known difficulties associated with recruiting low SEP individuals combined with the fact that only approximately 28% of the Dutch population is considered to have a low educational level [18], we included individuals with both low and medium educational level in the lower SEP group. Adults were considered low or medium SEP if their highest obtained educational level was primary education, intermediate vocational education, or higher secondary education. As shopping was done for the household, only 1 person per household was allowed to participate.

### Recruitment of Participants

According to the sample size calculation, at least 300 participants were needed to find a statistically significant difference in one of the main outcomes of the trial (vegetable purchases) between the control condition and experimental conditions (not yet accounting for possible attrition). Details regarding the sample size calculation can be found in Multimedia Appendix 1. Both traditional and more novel recruitment strategies were used to recruit participants. The traditional recruitment methods included advertising via flyers. The more novel recruitment strategy included using social media advertising, which has become an increasingly popular approach [11]. Flyers were distributed directly to participants on the street, at local events, and in real-world supermarkets. The flyers contained information on the inclusion criteria, activities within the study, and the reward for completing the study (a guaranteed incentive of €25 [~US $30]). Distribution of the flyers took place in October 2018. Flyers were also distributed around the University campus in October 2018. Approximately 500 flyers were printed at €0.35 (US $0.42) per flyer. Flyers were also delivered to addresses in low-income neighborhoods via postal services and by the study team. Low-income neighborhoods (ie, those with an average household income per resident under the median Dutch household income) were selected in order to increase participation rates of lower SEP individuals [19,20]. The social media campaign consisted of pay-per-click Facebook and Instagram campaigns and ran from mid-September to mid-December 2018. A professional was hired to set up the Facebook and Instagram campaigns. Campaigns were separated for low and high SEP target groups. Using existing and nondisclosed Facebook algorithms, the campaigns were adapted automatically based on what worked best for each target group. The target groups of the 2 Facebook campaigns were adjusted according to the characteristics of participants included in the study at a particular point in time. For example, if too few men had been recruited for the study after a few weeks, the Facebook campaign was adjusted to only include men in order to increase the recruitment of men. In addition, a Twitter post was created using the Supreme Nudge account (with over 250 followers at that time). Participants recruited from Facebook, Instagram, and Twitter were considered to be recruited using “social media strategies.” Although the researchers did not actively encourage participants to recruit others (eg, there was no incentive for participants to recruit others for the study), participants were also recruited by word-of-mouth at no cost to the researchers. A log to track the costs of the different recruitment strategies was kept by the study team. Furthermore, a log tracking the development of recruitment material (eg, posters and Facebook campaign) by the researchers and the distribution of posters by the researchers was kept. We intended to recruit participants between September and December 2018. If insufficient participants completed the study within this period (N≤300), recruitment would have continued in January 2019.

### Study Procedure

The social media campaigns and the study flyers directed potential participants to a registration website where more information about the study was provided and visitors could be redirected to a Survalyzer questionnaire for informed consent by entering their email address. Potential participants received an email with a link to the baseline questionnaire, which included questions regarding their sociodemographic characteristics and shopping habits. Inclusion criteria were assessed using the baseline questionnaire. If participants met the inclusion criteria, they received a link to the virtual supermarket and were asked to download the virtual supermarket to their computer and conduct a trial shop in which they needed to find 5 specific products from a grocery shopping list. Participants that successfully retrieved at least four out of five products were included in the study (Multimedia Appendix 1). Participants were then asked to shop 5 times in the virtual supermarket over the course of 5 consecutive weeks. During the first virtual shopping trip, participants were asked the following: “Imagine that you only have herbs at home and you decide to do the shopping for the entire household (people for whom you normally do the grocery shopping for) for one week. You receive a budget from us. You buy all your daily meals, snacks and drinks for the entire week (toiletries and alcohol are not for sale in this supermarket). The budget is only a guideline; it is possible to spend a little less or a little more.” For the subsequent 4 shopping trips, this prompt was updated to include the information that their usual supermarket was now closed and, as such, that they had to do their shopping in a new supermarket. Participants received guaranteed incentives for completing weekly shops: after participants completed their first shopping trip, they received a €5 (~US $6.05) gift voucher and after completing all 5 rounds, participants received an additional €20 (~US $24) gift voucher.

### Participant Characteristics

When assessing the eligibility of participants through a questionnaire, participants also answered questions regarding their age (years), sex (male or female), height (meters), weight (kilograms), household size (number of children and adults in the household), household net monthly income (ranging from <€1700 [~US $1815] to >€5000 [~US $6053]), highest educational level attained (primary school, secondary school, vocational education, or higher education), employment status (full-time employed, part-time employed, housewife/man, receiving benefits, retired, student, and other), responsibility for household shopping (fully responsible, mostly responsible, partly responsible, and someone else is responsible), frequency of household shopping (less than once a week, once a week, twice a week, three times a week, and more often), weekly budget for food shopping (<€25 [~US $30], €26-€50 [~US $31-US $61], €51-€100 [~US $62-US $121], €101-€150 [~US $122-US $182], €201-€250 [~US $243-US $303], €251-€300 [~US $303-US $363], and >€300 [>US $363]), and location of usual food shopping (at the market, in the supermarket, in small local shops, in organic food shops and other). After completing the final round of shopping, participants were also asked 8 questions regarding their experience of the virtual supermarket. Examples of prompts were “The program was easy to understand” and “The products I purchased in the virtual supermarket resemble my regular food purchases.” These items have been used in previous studies to assess participants’ experience of other virtual supermarkets [6,7]. Answering options were 5-point Likert scales ranging from “strongly disagree” to “strongly agree.” Results regarding participants’ experience of the virtual supermarket is presented in Multimedia Appendix 2.

### Outcome Measures

We collected data on participant characteristics and recruitment method to describe the type of participants that were recruited and retained using the different recruitment strategies. Furthermore, data on the costs associated with the different recruitment strategies were collected.

### Analyses

#### Overall Recruitment and Retention of Participants

We report descriptive statistics on the overall number of participants recruited and retained using the different recruitment strategies.

#### Recruitment Cost, Time, and Retention Rates According to Recruitment Strategy

Descriptive statistics on the number of participants recruited and retained using the different recruitment strategies and the costs associated with these strategies are reported. The cost per recruitment strategy was calculated by dividing the total amount spent on a recruitment strategy by the number of participants recruited via the corresponding strategy. This was also done for the time researchers spent on each recruitment strategy.

#### Participant Characteristics

Differences in participant characteristics between those who signed up for the study and met the inclusion criteria, participants that successfully conducted a training shop, participants that completed the study (ie, carried out all 5 rounds of shopping), and study noncompleters were inspected visually and formally tested. Differences between completers and noncompleters and differences in population characteristics between the different recruitment strategies were assessed using a one-way ANOVA for continuous outcome variables (ie, BMI and age) and the Pearson chi-square test in the case of categorical outcome variables (eg, educational level and income). Non-normally distributed continuous outcome variables were log transformed. Analyses were conducted in STATA version 14.1 (StataCorp) and a *P*-value of .05 was used to indicate statistical significance.

## Results

### Overall Recruitment and Retention of Participants

**Figure 1:**
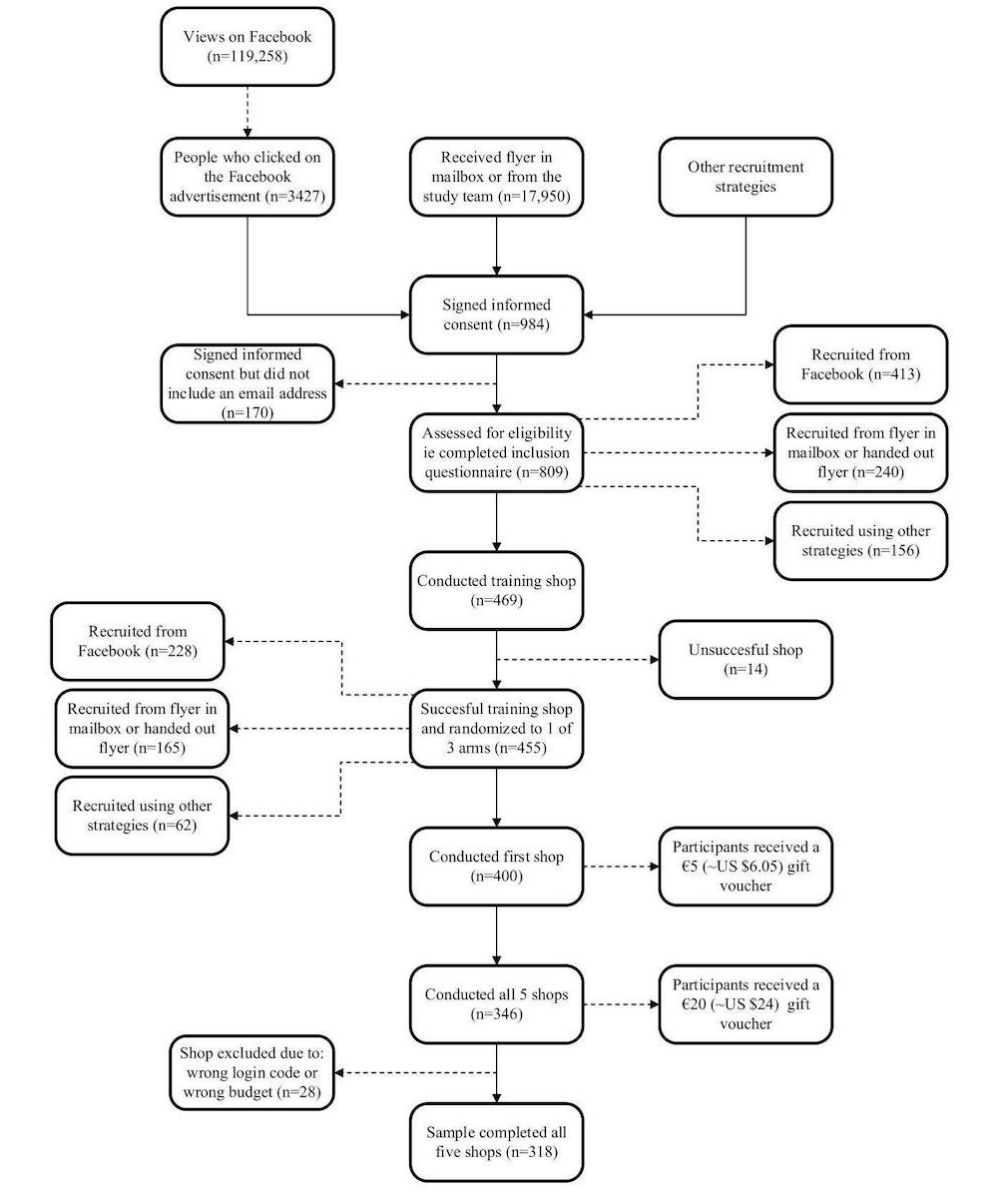
Flow chart of participant recruitment and retention.

Participants were recruited between September and December 2018. Figure 1 shows the flow of participants over the trial period. Regarding the recruitment campaigns, 3427 people clicked on the advertisement and were directed to the registration website. Initially, the campaign was much more likely to reach women (often aged 45 and older), after which the campaign was adapted to reach more men. This resulted in the campaign mostly reaching men under the age of 25. Around 17,500 people received a study flyer in their mailbox and 450 people received a study flyer directly from the study team. After being recruited by the different strategies and registering via the website, 809 people provided informed consent and were eligible for participation. Of those, 455 successfully conducted the training shop and were included in the study. A total of 346 participants completed the study and 318 participants generated usable data for all 5 rounds of shopping (ie, generated data that could be linked back to the participant and in which the login code corresponded to the participants’ assigned budget). Of all participants who successfully conducted the training shop, 76.0% (346/455) completed the study.

### Recruitment Cost, Time, and Retention Rates According to Recruitment Strategy

In Table 1, the costs of various recruitment strategies and the number of participants recruited using the different recruitment strategies are displayed. Over half (n=426) of the 809 adults who signed up for the study were recruited using the social media campaigns (mostly Facebook) and 29.7% (n=240) were recruited using flyers distributed to home addresses. Social media campaigns resulted in the highest absolute registration, inclusion in the study, and study completion. Distribution of flyers by the research team was the most expensive strategy, while the social media campaigns were the least expensive paid strategy in terms of the cost per person after completion of the study. Regarding the Facebook campaign, the cost per click was estimated to be **€**0.14 (US $0.17). The unpaid recruitment strategy “word-of-mouth” required no time investment by the research team and involved no recruitment costs. Regarding paid recruitment strategies, social media campaigns were the most time-efficient and flyers distributed directly by the study team were the least time-efficient for the researchers (8 minutes per study completer compared to 100 minutes per study completer, respectively). While the costs of flyers distributed by the team were similar to flyers distributed to home addresses (ie, €21 [US $25.52] and €20 [US $24.21], respectively), the time investment for flyers distributed directly by the study team was much higher compared to flyers distributed to home addresses (ie, 100 minutes and 25 minutes, respectively). The highest study completion rate was achieved with the “word-of-mouth” recruitment strategy (from 6.8% [55/809] of registered participants to 11.6% [40/346] of study completers). These results are also confirmed when calculating the percentage of participants that were included and completed the study compared to those that registered for each recruitment strategy (Table 2). In total, 72.7% (40/55) of participants recruited via word-of-mouth completed the study, compared to 39.9% (170/426), 44.2% (106/240), 54% (27/50), and 3.4% (3/38) of participants recruited via social media campaigns, flyers distributed to home addresses, flyers distributed directly by the study team, and unknown recruitment strategies, respectively (Table 2).

**Table 1:** The cost, time, and percentage of participants in each recruitment strategy during three phases of the study.

Recruitment type	Cost (€^a^)	Time (min)	Registered				Included				Completed		
			n (%) (N=809)	Cost (€) per participant	Time (min) per participant	n (%) (N=455)		Cost (€) per participant	Time (min) per participant	n (%) (N=346)		Cost (€) per participant	Time (min) per participant
Social media campaigns	1.298	1440	426 (52.7)	3	3	235 (51.6)		6	6	170 (49.1)		8	8
Flyers distributed to home addresses	2.142	2700	240 (29.7)	9	11	131 (28.8)		16	21	106 (30.6)		20	25
Flyers distributed directly by the team	0.558	3000	50 (6.2)	11	60	38 (8.4)		16	71	27 (7.8)		21	100
Word-of-mouth	0	0	55 (6.8)	0	0	46 (10.1)		0	0	40 (11.6)		0	0
Unknown	N/A^b^	N/A	38 (4.7)	N/A	N/A	5 (1.1)		N/A	N/A	3 (0.9)		N/A	N/A

^a^€1 = Approximately US $1.2.

^b^N/A: not applicable.

**Table 2:** The percentage of participants that were included in the study and completed the study compared to those that registered in each recruitment strategy.

Recruitment type	Registered, N	Included, n (%)	Completed, n (%)
Social media campaigns	426	235 (55.2)	170 (39.9)
Flyers distributed to home addresses	240	131 (54.6)	106 (44.2)
Flyers distributed directly by the team	50	38 (76.0)	27 (54.0)
Word-of-mouth	55	46 (83.6)	40 (72.7)
Unknown	38	5 (13.2)	3 (7.9)

### Participant Characteristics

Characteristics of participants who signed up for the study and met the inclusion criteria, participants that successfully conducted a training shop, participants that completed the study (ie, carried out all 5 rounds of shopping), and study noncompleters are presented in Table 3. The median age of participants included in the study was 31 (SD 25.0) and the majority of participants were female. Most participants included in the study had a medium educational level and a monthly household net income below €1700 (~US $2057). Study completers were somewhat younger than study noncompleters (median age of 31.0 compared to 37.0, respectively), but this difference was not statistically significant (*P*=.21). Study noncompleters were statistically significantly more often overweight (*P*=.01) and had older computers (*P*≤.001) compared to study completers. No other large observable differences in participant characteristics were found between study completers and noncompleters. For study completers, the average time in days between participants’ first shop and last shop was 38.1 (SD 13.1).

Participant characteristics by recruitment strategy can be found in Table 4. The participant characteristics age, overweight status, employment situation, and the percentage of households with at least two adults differed statistically significantly by recruitment strategy (Table 4). For example, the average age of participants recruited via social media was lower, and a larger proportion of overweight or obese participants were recruited via flyers distributed to home addresses.

**Table 3:** Characteristics of the study population who completed and did not complete the study.

Characteristics				Total sample (N=809)		Sample included (N=455)		Completers (N=346)		Noncompleters (N=463)		*P* value
Median (IQR) age, years				35.0 (27.0)		31.0 (25.0)		31.0 (24.0)		37.0 (30.0)		.21
Female sex, n (%)				515 (63.7)		284 (62.4)		215 (62.1)		299 (64.6)		.53
Mean (SD) BMI^a^				25.3 (5.3)		24.9 (4.8)		24.9 (4.9)		25.6 (5.5)		.05
Overweight status, n (%): overweight or obese^a,b^				348 (43.8)		176 (39.9)		129 (38.9)		219 (48.8)		.01
**Educational level^c^, n (%)**												.07
	Low educational level		90 (11.1)		43 (9.5)		31 (9.0)		59 (12.7)			
	Medium educational level		379 (46.8)		212 (46.6)		158 (45.7)		221 (47.7)			
	High educational level		337 (41.7)		200 (44.0)		157 (45.4)		180 (38.9)			
**Monthly household net income^d^, n (%)**												.32
		€0-€1700^e^	306 (38.3)		172 (38.6)		123 (36.5)		183 (40.3)			
		€1701-€2500	195 (24.4)		105 (23.5)		81 (24.0)		114 (25.1)			
		€2501-€3500	140 (17.5)		84 (18.8)		69 (20.5)		71 (15.6)			
		More than €3501	159 (19.9)		90 (20.2)		70 (20.8)		89 (19.6)			
**Employment situation,** **n (%)**												.12
		Full time job	183 (22.6)		108 (23.7)		90 (26.0)		93 (20.5)			
		Part time job	206 (25.5)		112 (24.6)		84 (24.3)		122 (26.9)			
		Student	187 (23.1)		118 (25.9)		85 (24.6)		78 (17.2)			
		Unemployed^f^	204 (25.2)		101 (22.2)		78 (22.5)		126 (27.8)			
		Entrepreneur or other	29 (3.6)		16 (3.5)		9 (2.6)		20 (4.4)			
**Household composition,** **n (%)**												
		At least two adults	547 (67.6)		312 (68.6)		243 (70.2)		314 (69.2)		.40	
		At least one child	263 (32.5)		141 (31.0)		109 (31.5)		154 (33.9)		.60	
**Type of computer,** **n (%)**												.56
		Apple-based	106 (13.1)		54 (11.9)		44 (12.7)		62 (13.7)			
		Windows-based	495 (61.2)		293 (64.4)		213 (61.6)		291 (64.1)			
		Other or unknown	93 (11.5)		42 (9.2)		33 (9.5)		51 (11.2)			
		Two or more computers/laptops	115 (14.2)		66 (14.5)		56 (16.2)		59 (13.0)			
**Computer age in years,** **n (%)**												<.001
		Less than 3 years	411 (50.8)		245 (53.8)		183 (52.9)		228 (49.2)			
		Between 3 and 6 years	310 (38.3)		175 (38.5)		140 (40.5)		170 (36.7)			
		Older than 6 years	70 (8.7)		30 (6.6)		20 (5.8)		50 (10.8)			
		Unknown	18 (2.2)		5 (1.1)		3 (0.9)		15 (3.2)			

^a^14 missing values.

^b^Participants with a BMI higher than 25.0 were considered overweight or obese.

^c^Low educational level included participants with primary education, medium educational level included participants with lower or higher secondary education and high educational level included participants with tertiary education.

^d^Nine missing values.

^e^€1 = Approximately US $1.2.

^f^Includes those who are retired, unemployed, unable to work and/or receiving social benefits and housewives/husbands.

**Table 4:** Characteristics of the study population for the entire sample and stratified by recruitment strategy.^a^

Characteristics			Total sample (N=455)		Social media (N=235)		Flyers to home addresses (N=131)		Flyers from study team (N=38)		Word-of-mouth (N=46)		*P* value	
Median (IQR) age, years			31.0 (25.0)		25.0 (18.0)		46.0 (26.0)		39.0 (25.0)		27.0 (25.0)		.02	
Female sex, n (%)			284 (62.4)		131 (55.7)		92 (70.2)		26 (68.4)		32 (69.6)		.03	
Mean (SD) BMI^b^			24.9 (4.8)		24.7 (4.9)		25.4 (4.4)		24.7 (5.4)		24.3 (5.4)		.63	
Overweight status: overweight or obese status^b,c^, n (%)			176 (39.5)		87 (38.5)		65 (50.0)		10 (27.0)		11 (24.4)		.01	
**Educational level^d^, n (%)**														.22
	Low educational level	43 (9.5)		19 (8.1)		19 (14.5)		1 (2.6)		3 (6.5)				
	Medium educational level	212 (46.6)		111 (47.2)		63 (48.1)		16 (42.1)		22 (47.8)				
	High educational level	200 (44.0)		105 (44.7)		49 (37.4)		21 (55.3)		21 (45.7)				
**Monthly household net income^e^, n (%)**														.34
	€0-€1700^f^	172 (38.1)		100 (42.7)		38 (29.0)		15 (39.5)		17 (37.0)				
	€1701-€2500	105 (23.3)		45 (19.5)		39 (29.8)		11 (28.9)		8 (17.4)				
	€2501-€3500	84 (18.6)		41 (17.7)		26 (19.8)		5 (13.2)		11 (23.9)				
	More than €3501	90 (20.0)		45 (19.5)		28 (21.4)		7 (18.4)		10 (21.7)				
**Employment situation, n** **(%)**														.02
	Full-time job	108 (23.7)		50 (21.3)		35 (26.7)		9 (23.7)		13 (28.3)				
	Part-time job	112 (24.6)		48 (20.4)		31 (23.7)		14 (36.8)		18 (39.1)				
	Student	118 (25.9)		89 (37.9)		9 (6.9)		6 (15.8)		13 (28.3)				
	Unemployed^g^	101 (22.2)		42 (17.9)		50 (38.2)		5 (13.2)		2 (4.3)				
	Entrepreneur or other	16 (3.5)		6 (2.6)		6 (4.6)		4 (10.5)		0 (0.0)				
**Household composition, n** **(%)**														
	At least two adults	312 (68.6)		152 (64.7)		97 (74.0)		22 (57.9)		38 (82.6)		.01		
	At least one child	141 (31.0)		74 (31.5)		43 (32.8)		12 (31.6)		12 (26.1)		.93		

^a^Unknown recruitment strategy was not included in the analyses.

^b^Nine missing values; 6 missing values for social media and 1 missing value for the other strategies.

^c^Participants with a BMI higher than 25.0 were considered overweight or obese.

^d^Low educational level included participants with primary education, medium educational level included participants with lower or higher secondary education and high educational level included participants with tertiary education.

^e^Four missing values for social media.

^f^€1 = Approximately US $1.2.

^g^Includes those who are retired, unemployed, unable to work and/or receiving social benefits and housewives/husbands.

## Discussion

### Principal Findings

This study found that the recruitment strategy word-of-mouth involved zero costs, required no time effort on the part of the researchers, and yielded the highest study-completion rate. Of all paid recruitment strategies, the least expensive strategy was social media campaigns. Social media campaigns also yielded the highest absolute registration and completion rates. Sociodemographic characteristics such as age, sex, and overweight status varied with the recruitment strategy.

Effective recruitment approaches are those that lead to the creation of a representative and large enough sample of study participants [21]. The combination of different recruitment strategies resulted in the recruitment of a relatively diverse study population in the space of 3 months. Social media campaigns were the most cost-efficient paid recruitment strategy employed and word-of-mouth was free, required no time effort on the part of researchers, and yielded in the highest retention rates. These results are comparable to previous studies carried out among the general population that report on the effectiveness and costs of recruitment via social media campaigns and other more traditional recruitment strategies [11,15,22]. For example, a study by Frandsen et al [15] found that social media drew more interest and was more cost effective than traditional methods such as flyering at baseline. Also, a systematic review investigating the effectiveness of Facebook as a recruitment strategy found reduced costs, shorter recruitment periods, better representation, and improved participant selection compared to traditional recruitment methods [22]. Surprisingly, a comparable study investigating the effectiveness of online methods to recruit participants for a virtual supermarket study found Facebook advertisements to be less successful as a recruitment strategy than was anticipated [6]. The use of a guaranteed incentive in this study and other studies that have successfully used Facebook to recruit participants (eg, [13]) may provide an explanation for the difference in findings between this study and the aforementioned study. A guaranteed incentive is likely to attract more people than no incentive or a prize lottery, for example. Future studies investigating the efficacy of social media campaigns for the recruitment of participants could investigate the role of incentives alongside this strategy.

Participants recruited via social media were less likely to complete the study compared to those recruited by flyers and word-of-mouth. In this study, word-of-mouth was found to be surprisingly effective; 10.1% (46/455) of the study population was recruited via word-of-mouth without the researchers actively encouraging participants to recruit peers. A disadvantage associated with recruitment via word-of-mouth, or via the exclusive use of a single recruitment strategy in general, is that it may yield a demographically skewed study population [15]. Contrary to the previous research finding that only age varied by recruitment strategy [11], we found that other demographic variables such as household composition, overweight status, sex, age, and employment situation all varied by recruitment strategy. Overall, our results suggest that it is important to use several different recruitment strategies if the aim is to include a diverse population (eg, younger and older adults with low and high SEP) in a study. Similarly, a systematic review investigating strategies for the successful recruitment of young adults to healthy lifestyle programs found that single recruitment strategies are less effective than mixed strategies, as fewer participants were recruited and higher attrition rates were reported when using a single recruitment strategy exclusively [14]. Nevertheless, despite using several recruitment strategies and targeting the social media campaigns to people with specific characteristics (eg, SEP, age, and sex), the recruitment strategies did not result in a sample that perfectly represented the target population. Instead, the study included a slightly younger population with more females and more highly educated participants. Differences in sociodemographic characteristics between this study sample and the average Dutch population may have been caused by the inclusion criteria of the study such as being the primary shopper for the household (leading to inclusion of more female participants) and the type of recruitment strategy used (eg, younger people may be more likely to be recruited via Facebook).

The current study results also suggest that recruitment strategies directly involving people (ie, active recruitment strategies using word-of-mouth or flyers distributed by the study team) lead to higher retention rates compared to recruitment strategies that do not involve personal contact (ie, passive recruitment strategies using social media campaigns and flyers distributed to homes). By contrast, the reach of social media campaigns and flyers sent to homes was much larger compared to the other recruitment strategies used. Moreover, social media campaigns can be used to target certain groups that are underrepresented in the study sample [22]. As such, neither active nor passive recruitment strategies are necessarily superior to the other [14]. Rather, it appears to be important to use a combination of *both* strategies, as active recruitment methods enhance recruitment and retention rates, but also require the most resources. However, despite the higher attrition rates associated with recruitment by means of passive strategies, these strategies do seem to have a wider reach and require only limited resources (especially when using social media) as compared to active recruitment strategies.

### Strengths and limitations

A strength of this study is the use of different recruitment strategies (eg, Facebook and flyers), which led to the creation of a diverse study population in a relatively short period. Furthermore, a relatively high completion rate of 76.0% (346/455) was found; this is particularly interesting in light of the fact that participants were asked to conduct 5 rounds of shopping over the course of 5 consecutive weeks. A limitation of this study is the limited generalizability of the results. While this study successfully recruited a relatively representative sample using traditional and novel recruitment strategies within the specified timeframe, the same might not apply to different studies in different settings. For example, we do not know whether our recruitment efforts were successful because of the methods used, because of the type of study (virtual supermarket study) that participants signed up for, or because of the guaranteed incentive of **€**25 (US $30). Another limitation of this study is that the study population was self-selecting, which could have led to the creation of a nonrepresentative study population (eg, due to the inclusion of more highly motivated adults). This type of bias may be inherent to this type of research in a community-based setting in which participants, by definition, need to sign up for a study themselves rather than be recruited by a physician, for example. This self-selection bias could, for example, be quantified by comparing the sociodemographic characteristics of the study sample with the sociodemographic characteristics of adults who received the study flyers.

### Conclusion

Regarding paid recruitment strategies, social media campaigns, particularly via Facebook, were more cost-effective than other more traditional methods. The unpaid recruitment strategy “word-of-mouth” yielded the highest study completion rate and required the least amount of time and effort on the part of the researchers. Employing only 1 recruitment strategy may lead to the creation of a demographically skewed sample.

## Appendix

Multimedia Appendix 1 Details regarding the SN VirtuMart trial.

Multimedia Appendix 2 Participant experience of the SN VirtuMart trial.

## Abbreviations

SEP: socioeconomic position
