# Supplementary material for: Recruitment of Participants for a 3D Virtual Supermarket: Cross-sectional Observational Study
Source: JMIR Form Res. 2021 Feb 9;5(2):e19234. doi: 10.2196/19234 (PMC7902190; doi:10.2196/19234)
Supplement: Multimedia Appendix 1 [file formative_v5i2e19234_app1.docx]

## The virtual supermarket program

The Supreme Nudge VirtuMart (SN VirtuMart) described in this study was developed by a member of the study group (NvdL). The SN VirtuMart was adapted from an existing virtual supermarket [1]. NvdL changed the layout of the virtual supermarket to replicate an average Dutch Coop supermarket (i.e., a supermarket chain in the Netherlands, partnering in the larger Supreme Nudge project). The virtual version was designed such that it reflects a medium-sized supermarket where people can conduct their weekly shopping. The gaming development platform UNITY was used to construct the 3D computer-based virtual supermarket [1]. 3D models of food and beverages were created in Blender and were designed to replicate real products (e.g., branding, size, shape, color, and style of packaging). The nutrition information of products was not displayed within the SN VirtuMart: the front of the product was also used for the back of the product. In order to simulate real-life supermarket shopping experiences, common marketing, branding and promotion techniques as well as sounds and background noise were used. The SN VirtuMart could be downloaded using a zip file and unpacking this to install and open the SN VirtuMart program on either Windows or Apple computers.

### Functionalities and user controls

Functionalities included the ability to move forward, turn left and right, look around, bend, view a product and the price up close, view the physical shopping basket and view a list of products within the shopping basket. Participants could use their arrow keys to turn left and right, go forward and to bend. Additionally, the mouse could be used to change the camera’s orientation (to look around). Participants could view the functionalities at all times by pressing escape. Participants could directly select products by left-clicking on the product and put them in their basket or they could right-click on the product to view a close-up of a product and then select the product to go into the basket. Participants were able to leave the virtual supermarket environment by walking to the cash register or pressing escape and choosing the option to leave the supermarket.

### Nudges within the SN VirtuMart

We implemented salience nudges to stimulate the purchases of healthier products and the substitution of unhealthier products for healthier ones. The salience nudges included bright orange frames around healthy low fat dairy products, a frame around the door of the frozen fruits and vegetables and orange arrows pointing from unhealthy to healthier high fiber variants.

### Prices and budget within the SN VirtuMart

Food prices, food labels and food placing could be adapted in Unity via Excel or a text editor with the aim to create different research conditions. Participants’ shopping budgets were based on self-reported real-life shopping budgets and implemented in the SN VirtuMart. Participants needed to spend at least 50% of their allocated budget in order to prevent participants from purchasing just a few items and quit the experiment. Participants could also overspend to a maximum of 125% to allow for overspending in the taxing arms [2]. Login codes were used to assign participants to certain conditions and budgets. Each week, during five consecutive weeks, participants received a new log in code. The log in codes were connected to a specific virtual shopping budget and a specific condition (e.g. control, nudging or pricing condition) within the virtual supermarket.

### Data collected in the SN VirtuMart

The virtual supermarket application stored information on time spent in the supermarket, participants’ walking routes through the supermarket, what products were looked at up-close, what products were placed into the shopping basket, what products were ultimately purchased and the total amount of money spent during a shop. Data was stored on both the participants’ computer as well as on the university server. Data was stored and sent to the server after participants clicked on the ‘leave supermarket’ button.

## Selection of food and beverage products

The SN VirtuMart included 1179 unique name-brand and budget-brand products categorized into 12 large food groups. Nonfood items and alcoholic beverages were excluded from the virtual supermarket. The SN VirtuMart did not include all food products that are normally present in a supermarket because it is not feasible to model all these products. Within each food category we selected top-selling products from an average Coop supermarket to be included in the stock of the virtual supermarket. The quantity and variety of products was such that participants with a variety of household sizes and budgets were able to do their weekly shopping in the virtual supermarket. Usual prices (i.e., excluding offers) for the selected products were collected from the Coop supermarket website in the summer of 2018.

## Study design

This study included a multi-period mixed study design consisting of three experimental arms (between-subjects design) and five experimental conditions (within-subjects design). The five experimental shopping conditions were: control, nudging, pricing, price salience and price salience with nudging. The order in which the participants received the conditions was randomized. The three study arms were exposure to subsidies, taxes and subsidies and taxes. Each week, during five consecutive weeks, participants received a new log in code which they could use to log into the virtual supermarket. The log in codes were connected to a specific virtual shopping budget and a specific condition (e.g. control, nudging or pricing condition) within the virtual supermarket. The shopping budget was based on participant’s actual shopping budget. Participants needed to spend at least 50% of their allocated budget in order to prevent participants from purchasing just a few items and quit the experiment. Participants could also overspend to a maximum of 125% to allow for overspending in the taxing arms [2].

## Sample size

A sample size calculation for a linear mixed model with three between-subjects factors and four within-subject factors (the control and nudging conditions should be equal across the three arms and therefore count as one within-subject factor) using delta values of vegetable purchases as an effect size was conducted. Assuming that purchases translate to intake, the baseline vegetable intake was set at 900 grams per week with a standard deviation of 370 for all conditions based on previous literature [3]. Furthermore, we hypothesized that the target differences would be largest in the taxing and subsidy arm, that the differences for nudging would be smallest, that the price salience condition would have a larger difference than pricing alone and that the combination between price salience and nudging would lead to the largest difference with the control condition in increasing vegetable purchases. Based on these assumptions and previous research [4, 5], we filled in the values found in Table 1. A sample size of 50 participants per arm, leading to 150 participants in total was needed with 90% power and an alpha of 0.05. Because we also aimed to stratify for low and high SEP, the aim was to include 300 participants.

***Table 1. The hypothesized purchase and consumption of vegetables in grams per week according to study condition and arm as input for the sample size calculation***

| **Condition** | **Price increases**  **Mean (SD)** | **Price decreases**  **Mean (SD)** | **Price increases and decreases**  **Mean (SD)** |
| --- | --- | --- | --- |
| Control | 900 (370) | 900 (370) | 900 (370) |
| Nudges | 1035 (370) | 1035 (370) | 1035 (370) |
| Pricing | 1130 (370) | 1100 (370) | 1160 (370) |
| Price salience | 1160 (370) | 1130 (370) | 1190 (370) |
| Price salience and nudges | 1240 (370) | 1200 (370) | 1280 (370) |

# References

1. Van der Laan LN, Papies EK, Ly A, Smeets PA. How health goal priming promotes healthy food choice: a virtual reality fMRI study. Submitted. 2019.

2. Waterlander WE, Jiang Y, Nghiem N, Eyles H, Wilson N, Cleghorn C, et al. The effect of food price changes on consumer purchases: a randomised experiment. Lancet Public Health. 2019 Aug;4(8):e394-e405. PMID: 31376858. doi: 10.1016/S2468-2667(19)30105-7.

3. Van Rossum CTM, Buurma Rethans EJM, Vennemann FBC, Beukers M, Brants HAM, de Boer EJ, et al. The diet of the Dutch : Results of the first two years of the Dutch National Food Consumption Survey 2012-2016. RIVM, 2016.

4. Waterlander WE, Steenhuis IH, de Boer MR, Schuit AJ, Seidell JC. The effects of a 25% discount on fruits and vegetables: results of a randomized trial in a three-dimensional web-based supermarket. Int J Behav Nutr Phys Act. 2012 Feb 8;9:11. PMID: 22316357. doi: 10.1186/1479-5868-9-11.

5. Cawley J, Hanks AS, Just DR, Wansink B. Incentivizing nutritious diets: a field experiment of relative price changes and how they are framed National Bureau of Economic Research. 2016.
