# Supplementary material for: Recruitment of Participants for a 3D Virtual Supermarket: Cross-sectional Observational Study
Source: JMIR Form Res. 2021 Feb 9;5(2):e19234. doi: 10.2196/19234 (PMC7902190; doi:10.2196/19234)
Supplement: Multimedia Appendix 2 [file formative_v5i2e19234_app2.docx]

***Figure 1. Usability and appreciation of the virtual supermarket for participants who completed all five shops (n=346)***
